# Effects of a probiotic soy product and physical exercise on formation of pre-neoplastic lesions in rat colons in a short-term model of carcinogenic

**DOI:** 10.1186/1550-2783-6-17

**Published:** 2009-08-06

**Authors:** Maicon F Silva, Kátia Sivieri, Elizeu A Rossi

**Affiliations:** 1 UNESP São Paulo State University, Department of Food and Nutrition, Araraquara Rodovia Araraquara/Jau Km 1,14801-902, Araraquara, SP, Brazil

## Abstract

**Purpose:**

In this study the influence of moderate or intense physical exercise, alone or in combination with the consumption of a soya product fermented with *Enterococcus faecium*, on the development of colon cancer induced chemically in rats with 1,2-dimethylhydrazine (DMH), was investigated.

**Methods:**

Eighty male Wistar SPF rats were randomly allocated to 8 groups (n = 10). One week after the start of the program of product ingestion and/or physical activity, all animals except the controls (group I) were injected subcutaneously with 50 mg/kg b.w. of 1,2-dimethylhydrazine (DMH). This procedure was repeated at the end of the second week. At the end of the 6-week experiment, all the animals were euthanized; the colons were removed and numbers of ACF was estimated.

**Results:**

Twenty-four days after the induction of pre-neoplastic lesions, it was evident that the formation of ACF was not significantly reduced by the ingestion of the fermented product, by intense or moderate physical activity or by a combination of these factors, in comparison with the positive control group of rats (p < 0.05). On the other hand, the performance of intense exercise, on its own, increased the number of ACF.

**Conclusion:**

The results reported in this article show that consumption of the fermented soy product described here and the practice of physical exercise (intense or moderate) were incapable, separately or combined, of inhibiting the formation of ACF in DMH-induced rats. The intense physical exercise led to an increased number of foci in the colons of these rats and, probably, to greater susceptibility to colorectal cancer.

## Background

Carcinogenesis is a complex process involving events at several levels of organization, including molecular, cellular and morphological. It can be divided into three main phases: initiation, promotion and progression [1]. Specifically in colorectal cancer, the initiation phase can be recognized by the formation of lesions in the bowel called aberrant crypt foci (ACF), which can develop into cancerous tissue [2,3]. Such lesions have often been used as biomarkers of the initial phase of colorectal cancer in rats induced with 1,2-dimethylhydrazine (DMH) [4,5].

The etiology of cancer is still much under discussion, but it is already known that certain identifiable factors are almost always involved in malignant neoplasms of a given type and, in the case of colon cancer, a close correlation has been found with genetic predisposition, environmental factors and lifestyle [6]. Control of the body weight and engagement in physical exercise have been stressed as factors protecting against colon cancer [7-10], while smoking, alcoholic drinks and fatty, fiberless diets are seen as risk factors.

Concerning physical exercise, several authors point out that a regular work-out would stimulate peristalsis in the gut, diminishing the transit time of feces passing through the large intestine and thus the time of contact between carcinogenic substances formed with the fecal residue and the mucous membrane of the colon [11,12]. Conversely, a sedentary lifestyle would be associated with an increased risk of colon cancer in men and women [8].

Fermented food is an important component of traditional diets, both for its nutritional value and its prophylactic and therapeutic properties [13]. However, its consumption in Brazil remains at a low level, due probably to the relatively high price of such products [14].

Research has also demonstrated that the commensal lactic acid bacterium from the human gut, *Enterococcus *(formerly *Streptococcus) faecium *CRL 183, if consumed in a fermented soy product, has several beneficial effects on the health. These include appreciable cholesterol-reducing activity, stimulation of the immune system, anticarcinogenic activity and inhibition of post-menopausal osteoporosis [15-19].

In view of the possible benefits of ingesting *E. faecium *and the potential role of physical exercise in the prevention of certain types of cancer, we decided to test the effects of consuming soy product fermented with *E. faecium *CRL 183, while engaging (or not) in physical exercise (moderate or intense), on the formation of ACF in rats injected with DMH.

## Methods

### Animal maintenance and administering of products

Eighty 4-week-old male Wistar SPF rats, average weight 200 g, were obtained from the central animal facility at the State University of Campinas (CEMIB, UNICAMP-SP, Brazil). The animals were housed for 8 weeks in boxes within a vivarium cabinet (Alesco^®^, Brazil) equipped with air filtration, controlled temperature (22 ± 1°C) and a dark:light cycle of 12:12 h. During the experiment, the rats had free access to sterile water and sterilized commercial rat chow (Purina^®^, Brazil), with the following composition: 23% protein, 49% carbohydrate, 4% fat, 5% fiber, 7% ash and 6% vitamin C. The products being tested were administered daily by gavage, at 3 mL/kg body weight (b.w.) per day, throughout the 8-week period. All animal procedures were submitted to the Research Ethics Committee of the School of Pharmaceutical Sciences, UNESP at Araraquara (SP, Brazil), who approved the experimental protocol.

### Experimental design

The animals were randomly allocated to 8 groups (n = 10), which received the following treatments: Group 1 (control): Healthy rats that did not consume the fermented product or perform physical exercise; Group 2 (Positive control): Rats with induced colon cancer, not given fermented product or physical exercise; Group 3: Rats with induced colon cancer that consumed fermented product and performed moderate exercise; Group 4: Rats with induced colon cancer that consumed fermented product and performed intense exercise; Group 5: Rats with induced colon cancer that consumed fermented product but performed no physical exercise; Group 6: Rats with induced colon cancer that performed moderate exercise but consumed no product; Group 7: Rats with induced colon cancer that performed intense exercise but consumed no product and Group 8: rats with induced colon cancer that consumed unfermented product and performed no exercise.

### Production of fermented product

The fermented soy product was processed by the method described in [15]. The soy-based medium was inoculated with overnight cultures in milk of *Enterococcus faecium *CRL 183 (probiotic strain) (1.5% v/v) and *Lactobacillus helveticus *ssp. *jugurti *416 (1.5% v/v). The "yogurt" used in the experiment was prepared freshly each week and kept refrigerated (~5°C) throughout the period of ingestion by the rats. The viability of *E. faecium *CL183 was analyzed in each batch of fermented product, by serial dilution and colony-counting on M17 agar plates (Difco).

### Production of unfermented product

The composition of the unfermented soy product was identical to that of the soy product except that no bacterial inoculum was added and no fermentation performed. This product was acidified by adding sufficient lactic acid to match the pH of the fermented product (4.5).

### Physical exercise

The animals were induced to run for 1 hour a day on powered treadmills for rats (model EP 131, Insight, Brazil), set at 3–5% inclination, by the method described by [20]. The velocity was set at 355 m/min for intense activity and 17–20 m/min for moderate activity.

### Chemical induction of colon cancer

One week after the start of the program of product ingestion and/or physical activity, all animals except the controls (group I) were injected subcutaneously with 50 mg/kg b.w. of 1,2-dimethylhydrazine (DMH) (Sigma, St. Louis, USA), a chemical inducer of carcinogenesis in the colon, dissolved an aqueous solution of 1 mM EDTA (pH 6.5). This procedure was repeated at the end of the second week [5].

### Morphological analysis

At the end of the 6-week experiment, all rats were weighed and euthanized in a CO_2 _chamber [21]. Immediately, the colon was removed from each animal by ventral incision, from the proximal end to the rectum. It was washed with 0.9% NaCl solution to remove the feces, slit longitudinally and laid open on blocks of expanded polystyrene. These were immersed in 10% buffered formaldehyde solution for 48 h and then transferred to 70% aqueous ethanol [22]. The fixed colon segments were stained in 0.1% methylene blue solution for about 10 min. Starting at the distal end, 25 consecutive fields were examined at 10× magnification under a microscope coupled to an image-capture system (Nikon^®^, Japan), and the images analyzed to identify and count the ACF, applying the criteria described in [2].

### Statistical analysis

Data were processed by the SIGMASTAT program. Analysis of variance (ANOVA) and the post-hoc Tukey' test were used to look for differences between experimental groups in mean of ACF. Differences were declared significant when p < 0.05.

## Results

The mean number of ACF counted per 25 microscope fields in each group of rats is reported in Table 1. Foci with 2 or more aberrant crypts were counted. No ACF were seen in the uninduced rats (group 1). The largest number of ACF was seen in group VII, consisting of animals subjected only to intense exercise, and this number was significantly greater than the mean for group II (positive controls). On the other hand, group VII did not differ from groups IV (induced rats that consumed the "yogurt" and carried out intense exercise) or V (induced rats that consumed the "yogurt" but were not exercised). The remaining groups did not differ from each other (p < 0.05).

**Table 1:** Numbers of aberrant crypt foci (ACF)

**Groups**	**ACF**
**1**	0.00
**2**	1.60 ± 0.57 ^a^
**3**	2.00 ± 0.0^a^
**4**	3.20 ± 0.50^ac^
**5**	2.80 ± 0.50^ad^
**6**	2.00 ± 0.95^a^
**7**	3.80 ± 1.29^bcd^
**8**	1.16 ± 0.57^a^

Values are expressed as means ± S.D. (*n = 10 rats per group*). Values with the same letters are not significantly different by *post hoc *Tukey test at p < 0.05.

**Group 1**: healthy animals that did not receive the fermented product; **Group 2**: animals initiated with chemical carcinogen that did not receive the fermented product; **Group 3**: animals initiated with chemical carcinogen that received the fermented product plus moderate physical exercise; **Group 4**: animals initiated with chemical carcinogen that received the fermented product plus exhaustive physical exercise; **Group 5**: animals initiated with chemical carcinogen that received the fermented product; **Group 6**: animals initiated with chemical carcinogen that did moderate physical exercise; **Group 7**: animals initiated with chemical carcinogen that did exhaustive physical exercise; **Group 8**: animals initiated with chemical carcinogen that received the non-fermented product.

## Discussion

Many of the commonest cancers develop as a result of an interaction between endogenous and environmental factors, most notably the diet. It was reported in an epidemiological study [23] that 35% of all types of cancer are thought to be included inadequate diet among these causal factors. According to Tanaka [24], epidemiological and experimental studies have revealed that several micronutrients may have cancer preventing properties in several organs, including the large bowel. Most of these compounds are antioxidants, which might provide an explanation for these properties.

Our research group has investigated the correlation between the level of immunological signals (cytokines) and the capacity of a soy product, fermented with *E. faecium *CRL 183 and supplemented with calcium, to delay the development of colon cancer. In a long-term study (8 months) of rats, the highest levels of IL-4 and TNF-α were found in the groups that showed the lowest numbers of adenocarcinomas in response to DMH induction. The increased production of IL-4 probably had a controlling effect on the inflammatory process, delaying the development of tumors in the phase of progression [25].

Physical activity is associated with a reduced risk of colon cancer and biologically plausible mechanisms underlying this association are known, e.g. alterations in local prostaglandin synthesis, increasing intestinal mobility and decreased gastrointestinal transit time, resulting in shorter contact time between the colon mucosa and potential carcinogens [26]. According to Venditi [27] the risk of colon cancer is 40 to 50% lower in active than in sedentary individuals.

Chemoprevention, a novel approach for controlling cancer, involves the use of specific natural products or synthetic chemical agents to reverse, suppress, or prevent premalignancy before the development of invasive cancer. Several natural products, including grains, nuts, cereals, spices, fruits, vegetables, beverages, medicinal plants and herbs, and their various phytochemical constituents, including phenolics, flavonoids, carotenoids and alkaloids, as well as organosulfur compounds, have been suggested to confer protective effects against a wide range of cancers, including colon cancer [28].

The present study was designed to assess whether ingestion of a product fermented with *E. faecium *CRL 183, alone or in combination with moderate or intense physical exercise, might have an effect in the short term on carcinogenesis induced in rats. It that tests showed that thefermented product in question had a viable count of 10^7 ^CFU/mL of *Enterococcus faecium *in every processed batch used in the experiment and may thus be considered probiotic. Gonzales [29] reported that bacteria in fermented milk are capable of modifying the intestinal flora of a host only if they reach a population density of at least 10^7 ^CFU/g in the gut.

The initiation phase of carcinogenesis starts in the period of DMH injection and lasts for about 100 days. During this phase, aberrant crypts, which are morphologically abnormal variants of the crypts normally found on the mucous membrane of the colon, are monitored. Epithelial cell proliferation and aberrant crypt foci (ACF) have been used for early detection of factors that influence colorectal carcinogenesis in rats and can be induced by the colon carcinogen dimethylhydrazine (DMH). This efficient animal-tumor model could be a useful approach to studying the influence of exercise during the initiation and post- initiation period, and has already contributed to current understanding of colon carcinogenesis [30]. These pre-neoplastic lesions are considered to be highly relevant biomarkers [31,32]. ACF assays are often used to detect factors that could influence the initiation phase of carcinogenesis in the colon [33].

Our results showed that the ingestion of the fermented soy product (group 5) did not inhibit the development of ACF, indicating that this product was unable to impede the clonal proliferation of cells initiated by DMH in the intestinal mucosa, under these experimental conditions. There have been few investigations of possible correlations between colon cancer phases and probiotic consumption. However, Leblanc [34] observed that ingestion of yogurt, fermented with *Lactobacillus delbrueckii *ssp *bulgaricus *and *Streptococcus thermophilus*, did not retard the initiation phase of colon cancer in rats, but was able to inhibited promotion and progression of experimental colorectal cancer. According to the same authors, yogurt possesses a capacity to modulate the immune system by stimulating the production of cytokines such as TNF-α and IFN-γ, whose concentrations need to be raised for a carcinogenesis-controlling effect to be observed. However, in the study cited, the measured concentrations of these cytokines remained very low after 1–3 months of yogurt consumption. Our research group has investigated the capacity of an *E. faecium *CRL 183 pure suspension and a product fermented with the same microorganism in delay the development of colon cancer in a long-term study. The soy product did not inhibited the development of ACF at the end of experimental period; however, the animals that ingested the suspension of *E. faecium *CRL 183 showed a 50% decrease in the average number of tumors and a reduced formation of ACF [25].

In the present study, intense exercise (groups 4 and 7) was shown to be closely correlated with raised numbers of ACF found in animals chemically induced with DMH, compared to the control group that were induced but did no exercise. Mechanisms to explain how intense physical activity could accelerate the initiation of carcinogenesis have not been fully elaborated in published form. One possibility is that the associated high level of oxidative stress and depression of the immune system could facilitate the development of colon cancer [27]. Exhaustive exercise can promote the generation of free radicals, which in turn modify molecular components of the cell such as DNA and proteins [35].

Studies to date suggest that exercise can exert its cancer-preventive effects at many stages during the process of carcinogenesis, including both tumour promotion and progression [36]. Among the possible mechanisms offered to explain this observation are the speeding up of the transit of material through the alimentary canal, strengthening of the immune system, changes in bile metabolism and altered levels of prostaglandin [37].

Exercise may alter tumour initiation events by modifying carcinogen activation, specifically by enhancing the cytochrome P450 system and selective enzymes in the carcinogen detoxification pathway, including, but not limited to, glutathione-S-transferases. Furthermore, exercise may reduce oxidative damage by increasing the level of a variety of anti-oxidant enzymes, enhancing DNA repair systems and improving intracellular protein repair systems [38,39].

Regarding moderate exercise, our results showed that this also failed to inhibit the development of ACF in the initial phase of carcinogenesis, contradicting the vast majority of published data, which shows a strong positive correlation between colon cancer and moderate physical exercise [12,30,35,37,38]. However, in many cases the experimental period of the physical exercise is longer than 12 weeks [40-43], whereas in our study the period was only 6 weeks. On the other hand, many models with induced colon cancer use a 20 at 40 mg/kg of DMH [30,44,45], while in the present work 50 mg/kg of DMH was used. This could have masked the potential beneficial effect of physical exercise.

The mechanisms underlying the exercise-induced protection against pre-neoplastic lesions are still not clear. It has been suggested that calorie restriction-induced weight loss and an exercise-induced negative energy balance inhibit the initiation or proliferation of ACF on the colon mucosa [46]. However, the present study the body weight gain was not significantly reduced by training of any intensity and all animals received a controlled feed and none showed signs of obesity.

The results reported in this article show that consumption of the fermented soy product described here and the practice of physical exercise (intense or moderate) were incapable, separately or combined, of inhibiting the formation of ACF in DMH-induced rats. In fact, intense physical exercise led to an increased number of foci in the colons of these rats and, probably, to greater susceptibility to colorectal cancer. Further research is needed, however, to have a better understanding of the complex interaction between the type of exercise and the phases (initiation, promotion and progression) of colon cancer.

## Competing interests

This study was supported by an internal research grant from UNESP University. The Principal Investigator (E.R) received remuneration from the UNESP University. None of the co-investigators (co-authors) received financial remuneration. All other researchers declare that they have no competing interests and independently collected, analyzed, and interpreted the results from this study.

## Authors' contributions

MS assisted in coordination of the study, data acquisition, in performing the statistical analysis, and drafting the manuscript. KS and ER participated in the data acquisition and drafting the manuscript. All authors have read and approved the final manuscript.
